# Prospective of Colorectal Cancer Screening, Diagnosis, and Treatment Management Using Bowel Sounds Leveraging Artificial Intelligence

**DOI:** 10.3390/cancers18020340

**Published:** 2026-01-21

**Authors:** Divyanshi Sood, Surbhi Dadwal, Samiksha Jain, Iqra Jabeen Mazhar, Bipasha Goyal, Chris Garapati, Sagar Patel, Zenab Muhammad Riaz, Noor Buzaboon, Ayushi Mendiratta, Avneet Kaur, Anmol Mohan, Gayathri Yerrapragada, Poonguzhali Elangovan, Mohammed Naveed Shariff, Thangeswaran Natarajan, Jayarajasekaran Janarthanan, Shreshta Agarwal, Sancia Mary Jerold Wilson, Atishya Ghosh, Shiva Sankari Karuppiah, Joshika Agarwal, Keerthy Gopalakrishnan, Swetha Rapolu, Venkata S. Akshintala, Shivaram P. Arunachalam

**Affiliations:** 1 Department of Internal Medicine, UChealth Parkview Medical Center, Pueblo, CO 81005, USAnbuzaboon@gmail.com (N.B.);; 2 Digital Engineering & Artificial Intelligence Laboratory (DEAL), Mayo Clinic, Jacksonville, FL 32224, USAsamiksha190404@gmail.com (S.J.); iqrajabeen65@gmail.com (I.J.M.); bipashagoyal@gmail.com (B.G.); patelsagar9799@gmail.com (S.P.); mohammednaveedshariff.r@gmail.com (M.N.S.); thangeswarann@gmail.com (T.N.); atishyaghosh@gmail.com (A.G.);; 3 Department of Internal Medicine, Zucker School of Medicine at Hofstra, Northwell Internal Medicine Residency at Vassar Brothers Medical Center, Poughkeepsie, NY 12601, USA; 4 Department of Internal Medicine, Carle Health, Urbana, IL 61801, USA; 5 Department of Internal Medicine, Mercy Catholic Medical Center, Darby, PA 19023, USA; agarwaljoshika@gmail.com; 6 Department of Internal Medicine, Wright Medical Center, Scranton, PA 18503, USA; 7 Medical Gastroenterology, AIG Hospitals, Hyderabad 500032, India; vakshin1@jhmi.edu; 8 Division of Gastroenterology & Hepatology, Department of Medicine, Johns Hopkins School of Medicine, Baltimore, MD 21287, USA; 9 Department of Critical Care Medicine, Mayo Clinic, Jacksonville, FL 32224, USA

**Keywords:** colorectal cancer, artificial intelligence, bowel sounds, gut sounds, phonoenterogram, bowel sound-based signal analysis, gastrointestinal tumor detection, non-invasive diagnosis

## Abstract

**Background:** Colorectal cancer (CRC) is the second leading cause of cancer-related mortality worldwide, accounting for approximately 10% of all cancer cases. Despite the proven effectiveness of conventional screening modalities such as colonoscopy and fecal immunochemical testing (FIT), their invasive nature, high cost, and limited patient compliance hinder widespread adoption. Recent advancements in artificial intelligence (AI) and bowel sound-based signal processing have enabled non-invasive approaches for gastrointestinal diagnostics. Among these, bowel sound analysis—historically considered subjective—has reemerged as a promising biomarker using digital auscultation and machine learning. **Objective:** This review explores the potential of AI-powered bowel sound analytics for early detection, screening, and characterization of colorectal cancer. It aims to assess current methodologies, summarize reported performance metrics, and highlight translational opportunities and challenges in clinical implementation. **Methods:** A narrative review was conducted across PubMed, Scopus, Embase, and Cochrane databases using the terms colorectal cancer, bowel sounds, phonoenterography, artificial intelligence, and non-invasive diagnosis. Eligible studies involving human bowel sound-based recordings, AI-based sound analysis, or machine learning applications in gastrointestinal pathology were reviewed for study design, signal acquisition methods, AI model architecture, and diagnostic accuracy. **Results:** Across studies using convolutional neural networks (CNNs), gradient boosting, and transformer-based models, reported diagnostic accuracies ranged from 88% to 96%. Area under the curve (AUC) values were ≥0.83, with F1 scores between 0.71 and 0.85 for bowel sound classification. In CRC-specific frameworks such as BowelRCNN, AI models successfully differentiate abnormal bowel sound intervals and spectral patterns associated with tumor-related motility disturbances and partial obstruction. Distinct bowel sound-based signatures—such as prolonged sound-to-sound intervals and high-pitched “tinkling” proximal to lesions—demonstrate the physiological basis for CRC detection through bowel sound-based biomarkers. **Conclusions:** AI-driven bowel sound analysis represents an emerging, exploratory research direction rather than a validated colorectal cancer screening modality. While early studies demonstrate physiological plausibility and technical feasibility, no large-scale, CRC-specific validation studies currently establish sensitivity, specificity, PPV, or NPV for cancer detection. Accordingly, bowel sound analytics should be viewed as hypothesis-generating and potentially complementary to established screening tools, rather than a near-term alternative to validated modalities such as FIT, multitarget stool DNA testing, or colonoscopy.

## Simple Summary

Colorectal cancer is a common and serious disease, but many people delay or avoid screening because current tests can be invasive, uncomfortable, or expensive. This review explores a new research idea: using bowel sounds—the natural noises made by the intestines—combined with artificial intelligence to support colorectal cancer screening, diagnosis, and management. With modern digital stethoscopes, wearable sensors, and computer algorithms, bowel sounds can be recorded and analyzed in ways that were not possible before. The authors aim to summarize what is currently known about bowel sound analysis, how artificial intelligence can detect subtle patterns linked to bowel disease, and whether this approach could one day complement existing screening methods. While this technology is still experimental and not ready for clinical use, it may open new research pathways for developing safer, more accessible, and non-invasive tools to support early detection and monitoring of colorectal cancer.

## 1. Introduction

Colorectal cancer (CRC) represents colon or rectal cancer, depending on where it first manifests. Although these two types of cancer are remarkably different from each other, they are frequently grouped [1]. CRC is the third most common cause of cancer worldwide, reported by WHO, accounting for 10% of all types of cancer reported globally. It mostly affects individuals aged 50 years and above. It is currently the second leading cause of cancer-related deaths worldwide, and the rising incidence and prevalence of CRC are concerning issues [2]. The American Cancer Society estimates that 154,270 new cases and 52,900 deaths due to CRC in the United States will occur in 2025 [3]. As per the Global Cancer Observatory (2022), Denmark, Norway, Hungary, The Netherlands, and Croatia had the highest age-standardized incidence rates (ASR) per 100,000 population: 48.1, 45.3, 44.2, 42.8, and 41.1, respectively [4].

The development of CRC is believed to begin with a mutation in the adenomatous polyposis coli (APC) suppressor gene, leading to benign polyps that may take years to decades to become malignant. Hereditary familial adenomatous polyposis (FAP) is associated with a point mutation in the genes leading to the excessive growth of malignant polyps in the bowel. Other predominant pathways related to CRC include chromosomal instability, microsatellite instability, and the CpG island methylator phenotype, all of which correlate with loss of heterozygosity, a lack of a DNA repair system, and silencing of essential genes [5]. The leading risk factor for the development of CRC is age, and the incidence of the disease rises significantly after the age of 50. Additional factors that increase the risk of CRC include hereditary factors, positive family history, particularly those with relatives diagnosed with CRC before the fifth decade of life, and chronic bowel inflammatory diseases like Crohn’s or ulcerative colitis (UC), which increase the risk of developing CRC by 2.5% and 3.7%, respectively. Various lifestyle-related factors, including sedentary lifestyle, obesity, and increased dietary fat consumption, increase the risk of CRC via the release of proinflammatory cytokines that lead to chronic colon and rectum inflammation. Red meat is a potential carcinogen, and consumption of alcohol and tobacco significantly increases the risk of CRC [6]. Although there have been substantial advancements in the knowledge and clinical capacity of clinicians to manage CRC, there are still substantial challenges related to morbidity and death in patients with CRC due to unavoidable recurrence and metastases of the cancer [7]. CRC exhibits a subtle presentation with minimal symptoms in early stages, leading to nearly two-thirds of patients presenting in advanced stages that ultimately result in high death rates in patients with CRC [8,9]. Early diagnosis of CRC is crucial to prevent serious complications like intestinal perforation due to obstructive ileus [10]. To lower the morbidity and mortality rate of CRC, early diagnosis of colorectal polyps is essential. Effective screening methods are critical because they help identify high-risk polyps and reduce the number of unnecessary polyp or adenoma resections. This, in turn, decreases medical consequences that may arise due to biopsies and also reduces the financial burden on patients [11]. Colonoscopy is one of the most common methods to screen for CRC. It has numerous benefits when compared to other screening methods because it happens at shorter intervals and also has good sensitivity [10]. But this procedure has several drawbacks; for example, it is expensive, invasive, has low patient compliance, and has a high risk of complications [7]. The fecal immunochemical test (FIT) is another popular, cost-effective, and non-invasive alternative to colonoscopy; however, FIT accuracy is limited, and its sensitivity for CRC is less than 70% [1]. Furthermore, imaging tests, such as abdominal ultrasonography, CT, thoracic roentgenography, and NMR, are only helpful in advanced localized lesions, indicating their poor efficiency in the early identification of cancer [5]. Therefore, CRC screening tools face challenges in the form of low compliance (colonoscopy), low test accuracy (FIT), and the limited diagnostic utility of imaging in early-stage disease.

Therefore, it is crucial to develop a screening method that may, in the future, complement established screening strategies pending rigorous clinical validation [12]. To address the existing challenges, there has been a surge in studies on utilizing bowel sounds (BS) as a new diagnostic method [13].

Current non-invasive colorectal cancer screening modalities primarily rely on stool-based biomarkers or imaging-based structural assessment. While fecal immunochemical testing (FIT) and multitarget stool DNA assays offer non-invasive options, they depend on intermittent biomarker shedding and exhibit reduced sensitivity for advanced adenomas and early-stage lesions. Similarly, radiographic techniques such as CT colonography detect established structural abnormalities but lack sensitivity for subtle, early mucosal, or functional changes. In contrast, bowel sound-based signal analysis represents a fundamentally different diagnostic approach, capturing continuous, physiology-driven motility-related signals rather than static structural or molecular snapshots. By leveraging artificial intelligence to extract subtle temporal and spectral features from gastrointestinal acoustics, bowel sound analysis has the potential to identify early functional disturbances that may precede detectable biomarker release or radiographic abnormalities.

Integrating AI use in bowel sound analysis or phonoenterography could emerge as a novel technique that may complement and lead to the development of a screening tool that is safe, affordable, and accessible compared to traditional screening methods [13].

The unmet clinical need in colorectal cancer screening lies in identifying early functional and physiological alterations before overt structural changes or biomarker positivity occur. Conventional grayscale ultrasound and cross-sectional imaging primarily assess anatomical abnormalities and are limited in detecting subtle motility or microenvironmental changes associated with early colorectal neoplasia. This approach aligns with emerging trends in multimodality imaging and AI-driven diagnostics, where non-visual bio-signals complement traditional imaging to enhance early detection and clinical translation.

The purpose of this review was to explore the potential of phonoenterography as a safe, cost-effective, and noninvasive screening tool for the detection of CRC. It aims to describe how AI models, including ML and devices like digital stethoscopes, can improve the interpretive accuracy of phonograms in the early detection of CRC. The paper also evaluates safety, accessibility, accuracy, and affordability by comparing it with conventional screening techniques such as colonoscopy, FOBT, and imaging tests. It attempts to assess the perspectives of patients and healthcare providers regarding the perceived benefits of AI-based screening tests in the routine screening of CRC. Lastly, we aim to identify the potential limitations associated with incorporating AI-based bowel sound analysis into daily practice and provide recommendations for future research to optimize AI-driven bowel sound analysis for CRC screening. Traditional CRC screening relies on colonoscopy, FIT, and CT colonography, which, though effective, are limited by invasiveness and compliance issues. Artificial intelligence (AI)–assisted bowel sound-based signal analysis has been explored as a potential non-invasive physiological marker, building on analytic frameworks previously applied in inflammatory bowel disease (IBD) research.

## 2. Methods

### 2.1. Literature Search Strategy

This study was designed as a scoping narrative review intended to map the existing literature, identify emerging themes, and highlight research gaps in AI-based bowel sound analysis rather than to provide a systematic quantitative synthesis.

A comprehensive, structured search was performed in PubMed, Embase, Scopus, Web of Science, and the Cochrane Library for studies published between January 1995 and September 2025. The search strategy incorporated both Medical Subject Headings (MeSH) and free-text keywords related to colorectal cancer, bowel sounds, phonoenterography, gastrointestinal acoustics, artificial intelligence, machine learning, deep learning, and non-invasive diagnosis. Boolean operators were applied to maximize search sensitivity.

To identify additional eligible studies, reference lists of relevant original articles and reviews were manually screened.

### 2.2. Eligibility Criteria

Studies were eligible for inclusion if they involved human participants and utilized AI-based or digital signal-processing approaches for the analysis of bowel or gastrointestinal sounds, particularly in the context of colorectal cancer, gastrointestinal motility disturbances, or partial obstruction. Only full-text, peer-reviewed, English-language articles were included. Studies were excluded if they were animal or in vitro investigations, conference abstracts, non-peer-reviewed material, or lacked AI, machine-learning, or signal-processing methodology.

### 2.3. Data Extraction

For each included study, data were systematically extracted regarding study authorship, year of publication, and participant characteristics. Information was also collected on the bowel sound-based acquisition method, including whether bowel sounds were recorded using a digital stethoscope, wearable sensor, or smartphone microphone, as well as the sampling duration and anatomical recording site. Details of the computational approach were extracted, including the type of AI algorithm used—such as convolutional neural networks, gradient boosting, transformer models, or hybrid frameworks—and the feature extraction methods applied, including spectrograms, Mel-frequency cepstral coefficients, spectral entropy, or sound-to-sound interval analysis. Validation strategies and diagnostic performance metrics, including accuracy, sensitivity, specificity, F1-score, and area under the ROC curve (AUC), were also documented.

### 2.4. Synthesis Approach

Because the included studies varied widely in methodology, data acquisition, and analytic frameworks, a formal meta-analysis was not feasible. Instead, a qualitative synthesis was conducted to identify overarching technological patterns, algorithmic performance trends, and potential translational implications for clinical use. To promote thematic clarity, the literature was organized into three conceptual categories: (1) bowel sound-based physiology studies exploring bowel-sound generation and alteration in health and disease; (2) AI algorithmic studies developing computational pipelines for bowel-sound detection, segmentation, and classification; and (3) clinical application studies assessing the diagnostic or screening value of AI-assisted bowel sound analysis for colorectal cancer or related gastrointestinal conditions.

### 2.5. Quality Appraisal

The methodological quality of included studies was assessed using an adapted version of the SANRA criteria. This appraisal emphasized the clarity of stated objectives, thoroughness of the literature search, coherence and rigor of the narrative synthesis, and transparency regarding study limitations. Applying these criteria allowed for a structured evaluation of the heterogeneous evidence base and supported the development of a coherent and integrative overview of current progress and remaining gaps in AI-driven bowel sound analytics for colorectal cancer.

Given the heterogeneity of study designs, signal sources, and clinical endpoints, a PRISMA-style systematic review and meta-analysis were not feasible and were therefore not pursued.

## 3. Physiology and Bowel Sound-Based Basis of Bowel Sounds in Colorectal Cancer

### 3.1. Normal Physiology of Bowel Sounds

Peristaltic and segmental contractions, generated by smooth muscle cells under the control of the enteric nervous system and interstitial cells of Cajal (ICC), propel and mix luminal gas and fluid. These mechanical interactions create pressure changes and turbulence within the gut lumen, which are transmitted through the abdominal wall as audible bowel sounds [14,15,16,17,18,19,20].

### 3.2. Frequency Range (100–2000 Hz)

The frequency range of 100–2000 Hz encompasses the typical bowel sound-based spectrum of normal bowel sounds and reflects the biomechanical interactions between intestinal contractions and luminal contents. Most bowel sound energy lies within this band, with lower frequencies (100–300 Hz) corresponding to high-amplitude, short-duration events—often originating from the colon—while higher frequencies (up to 2000 Hz) are commonly associated with gastric and proximal small intestinal activity [21,22].

This spectrum is clinically important because it enables discrimination of true gastrointestinal acoustic signals from environmental noise, supporting reliable bowel sound acquisition using digital and electronic auscultation devices [23,24]. The specific frequency content depends on motility patterns, luminal gas-to-liquid ratios, and anatomical location. For example, dominant frequencies near 100 Hz are common in the colon, whereas gastric sounds often exhibit peaks around 300 Hz [1,2]. Accurate capture of this frequency range is essential for objective assessment of gastrointestinal motility and forms the basis for AI-driven bowel sound-based signal analysis [24,25].

### 3.3. Regional Differences Between Small and Large Bowel

The small bowel exhibits frequent, rhythmic peristaltic and segmental contractions generated by distributed ICC pacemakers. Jejunal contractions are typically stronger and more propagative compared to the ileum, supporting rapid mixing and nutrient absorption [26,27]. Bowel sound-based output from the small bowel is generally lower in amplitude and occurs in the mid-frequency range (~300 Hz) [21,22], making it less prominent than gastric or colonic sounds.

Large intestine:

Colonic motility consists of less frequent but more forceful contractions, including high-amplitude propagated contractions (HAPCs) that underlie mass movements and defecation. A dominant pacemaker region likely located in the cecum contributes to slower, coordinated, and cyclic motor patterns, including retrograde rectal contractions aiding continence [28]. Colonic bowel sounds are typically higher in amplitude but lower in frequency (~100 Hz), especially in the right lower quadrant [22].

### 3.4. What Constitutes “Normal” Bowel Sound-Based Activity

Normal bowel sound activity consists of intermittent bowel sound-based events within the 100–2000 Hz range, with clear regional patterns reflecting physiological motility. Frequency range: Lower-frequency (~100 Hz) colonic sounds contrast with higher-frequency (~300 Hz) gastric/small bowel signals [21,22]. Regional activity: The stomach generates the most frequent sounds, followed by the colon; the small intestine is relatively quiet in healthy individuals [21,22,29].

Physiological mechanisms: Sound generation is driven by contractions orchestrated by the ENS and ICC pacemaker activity. Patterns vary with the migrating motor complex (MMC) during fasting and irregular phasic contractions in the fed state, both of which shape bowel sound-based output [25,30]. Colonic haustral activity and cyclic motor patterns produce distinct low-frequency signatures [28,31].

### 3.5. How Tumors Alter Motility and Luminal Flow

Colorectal cancer alters gastrointestinal motility through mechanical, neural, cellular, and inflammatory mechanisms.

#### 3.5.1. Mechanical Obstruction

As tumors enlarge, the lumen narrows, restricting gas and stool passage. This can lead to constipation, distension, colicky pain, and acute large bowel obstruction—a frequent emergency in CRC [32,33]. Distal tumors pose a higher obstruction risk due to smaller luminal diameter and firmer stool consistency [32,34,35].

#### 3.5.2. Disruption of the Enteric Nervous System

Tumor invasion damages submucosal and myenteric plexus neurons, impairing peristaltic coordination. Altered neurotransmitter signaling—including changes in galanin, acetylcholine, and nitric oxide—further disrupts neural motility regulation [36,37,38,39].

#### 3.5.3. Mucosal and Microenvironmental Changes

Loss of mucus-secreting goblet cells compromises lubrication and barrier function, contributing to dysmotility and localized inflammation [40,41,42]. Tumor-associated fibroblast activation and desmoplastic remodeling stiffen the bowel wall, exacerbating luminal narrowing [37,43].

#### 3.5.4. Bowel Sound-Based Correlates of Obstruction

Proximal to obstruction: Increased intraluminal pressure and exaggerated peristaltic contractions generate high-pitched, “tinkling” sounds due to turbulent gas–fluid interactions [32,44].

#### 3.5.5. Distal to Obstruction

The bowel becomes silent owing to a collapsed lumen and absent peristalsis [32]. Spectral analyses confirm that obstructed bowel sounds are longer in duration and exhibit higher dominant frequencies [45], although auscultation alone is not reliable for diagnosis [32,46].

#### 3.5.6. Correlation with the Degree of Obstruction/Inflammation

Despite measurable bowel sound-based alterations in obstruction, studies demonstrate weak and inconsistent correlations between bowel sound features and the severity of obstruction or inflammation [45,46,47,48]. Inter-observer agreement for auscultation is poor [46,47,49], and inflammatory markers do not translate into reproducible bowel sound-based changes [49,50,51].

### 3.6. Bowel Sound-Based Phenotyping and Potential Biomarker Role

**Figure 1:**
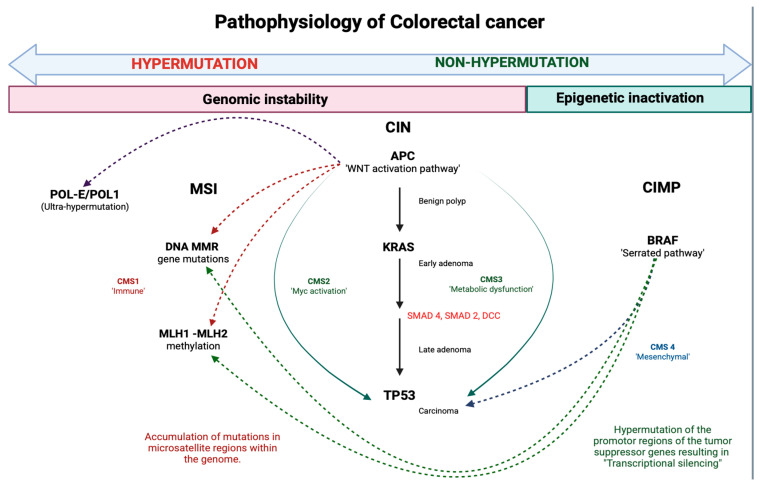
Colorectal cancer arises through three major molecular pathways: microsatellite instability (MSI) due to mismatch-repair defects, the chromosomal instability (CIN) pathway driven by APC → KRAS → TP53 mutations, and the CpG island methylator phenotype (CIMP) characterized by epigenetic silencing and frequent BRAF mutations. MSI tumors show hypermutation, CIN tumors follow the classic adenoma–carcinoma sequence, and CIMP tumors evolve from serrated lesions with widespread promoter methylation. Together, these pathways reflect the interplay of genomic instability and epigenetic inactivation in colorectal carcinogenesis.

Early-stage colorectal cancer (CRC) and large adenomatous polyps generally do not produce dramatic bowel sound abnormalities detectable by routine auscultation; however, subtle yet physiologically meaningful modifications in bowel sound-based features—such as sound intervals, entropy, spectral distribution, and amplitude—may occur due to early biomechanical, mucosal, and microenvironmental alterations. These changes form the conceptual basis for bowel sound-based phenotyping as a potential noninvasive biomarker for CRC. Early lesions—such as adenomatous polyps or intramucosal carcinoma—typically do not cause mechanical obstruction or major motility disturbances, so classic changes in bowel sounds (e.g., interval shortening, increased amplitude, or altered entropy) are not expected. However, subtle local effects may arise from early alterations in tissue stiffness, cellular architecture, and local inflammation, which can theoretically influence peristaltic patterns and micro-motility, leading to minor changes in sound intervals or complexity (entropy) [52]. CRC-associated bowel sound-based patterns are largely mediated through indirect effects such as partial obstruction, inflammation, and motility disturbance. [53,54]. Computerized analysis of bowel sounds (bowel sound-based phenotyping) has shown promise in distinguishing certain GI conditions, such as irritable bowel syndrome and post-operative ileus, by analyzing sound intervals, entropy, and amplitude. However, systematic reviews highlight that current evidence is limited, with small sample sizes and methodological weaknesses, and no validated bowel sound-based biomarkers exist for early colorectal neoplasia or polyps [48,55]. The sensitivity and specificity of bowel sound analysis for early-stage CRC or polyps remain unestablished, and routine auscultation is not recommended for screening or diagnosis [55,56,57,58]. These findings suggest that, in principle, similar subtle bowel sound-based changes could be detected in early neoplastic lesions, reflecting underlying pathophysiological alterations. However, current evidence indicates that while bowel sound-based phenotyping is feasible and can distinguish certain GI conditions, there is insufficient data to support its use as a reliable biomarker for early CRC or polyps. Most studies are limited by small sample sizes, methodological heterogeneity, and lack of validation in CRC populations [55]. The clinical value of bowel sound-based biomarkers for CRC detection is therefore unproven, and established screening relies on stool-based, serum, and molecular markers, as well as endoscopic evaluation [59,60,61,62,63]. Figure 1 below shows the pathophysiology of colorectal cancer.

## 4. AI and Technological Advances in Bowel Sound-Based Signal Analysis

Bowel sound-based signals referenced in colorectal cancer research arise from fundamentally different biological mechanisms and should not be conflated. Physiological bowel sounds reflect intraluminal gas–fluid movement and motility-driven turbulence. Photobowel sound-based signals originate from laser-induced thermoelastic expansion within tissue and represent ex vivo or imaging-based contrast mechanisms rather than spontaneous gastrointestinal acoustics. Wearable motility-monitoring systems capture indirect mechanical or vibrational correlates of movement rather than true bowel sounds. In this review, findings from each domain are discussed separately and are not extrapolated to CRC screening without explicit mechanistic justification.

### 4.1. Bowel Sound Acquisition Technologies

Technological advancements have transformed the acquisition of bowel sounds, leveraging contact microphones, piezoelectric sensors, electronic stethoscopes, wearable devices, and smartphone microphones.

Smartphone-based detection: Recent studies demonstrate that built-in smartphone microphones can reliably record bowel sounds, enabling non-invasive, accessible gut health monitoring. This approach offers a practical, scalable solution for remote or ambulatory monitoring [64].

#### 4.1.1. Convolutional Neural Networks (CNNs) vs. Long Short-Term Memory (LSTM) Networks

CNNs excel at extracting spatial and spectral features from short audio segments, making them highly effective for bowel sound event detection and segmentation. They have demonstrated high accuracy (often >90%) and sensitivity in both wearable and stationary systems [65,66].

LSTMs are designed to capture temporal dependencies and sequence patterns in audio data, which is valuable for modeling the irregular timing and duration of bowel sounds. However, in direct comparisons, CNNs generally outperform LSTMs for bowel sound recognition tasks, likely due to the short, burst-like nature of bowel sounds [64,67].

Hybrid models (e.g., ResNet-LSTM, CNN-transformer architectures) combine the strengths of both, enabling multifeature fusion and improved classification of bowel sound activity levels (normoactive, hyperactive, hypoactive) with superior accuracy and explain ability [67,68].

#### 4.1.2. BowelRCNN and Advanced AI Models

BowelRCNN refers to a class of models that integrate recursive (recurrent) neural networks with CNNs for bowel sound analysis. These hybrid architectures can capture both local bowel sound-based features and longer-term temporal patterns, achieving high specificity and supporting clinical diagnosis [69].

Other advanced models (e.g., YOLO-based, Branchformer, and transformer-augmented CNNs) further enhance real-time detection and classification, leveraging self-attention mechanisms and self-supervised pre-training to improve robustness, especially in data-limited scenarios [68,70].

#### 4.1.3. Technological Advances

Wearable devices and low-cost piezoelectric sensors enable long-term, ambulatory monitoring and real-time wireless transmission of bowel sound data [66,71].

Phonoenterography and electronic stethoscopes provide high-fidelity recordings for AI-driven analysis, supporting non-invasive, cost-effective GI diagnostics [13,68].

Spectral analysis and unsupervised grading systems offer objective quantification of motility and activity, supplementing traditional auscultation [25].

### 4.2. Signal Preprocessing and Feature Extraction

Signal preprocessing and feature extraction are critical steps in AI-driven bowel sound-based signal analysis, enabling accurate detection, classification, and interpretation of gastrointestinal activity. These processes transform raw audio signals—whether captured by contact microphones, electronic stethoscopes, or smartphone microphones—into meaningful features for machine learning models such as convolutional neural networks (CNNs), long short-term memory networks (LSTMs), and hybrid architectures.

#### 4.2.1. Signal Preprocessing Typically Involves

Noise reduction and filtering: Techniques such as wavelet-based filtering, autoregressive moving average models, and empirical mode decomposition are used to remove background noise and artifacts, isolating bowel sounds from other abdominal or environmental sounds [13,72].

Segmentation: Algorithms identify and extract bowel sound events from continuous recordings, often using amplitude thresholds, spectral changes, or time-domain features. Accurate segmentation is essential for downstream analysis and is a key focus in CNN-based detectors [64,65,66].

Normalization: Standardizing signal amplitude and duration ensure consistency across recordings and subjects.

#### 4.2.2. Feature Extraction Converts Preprocessed Signals into Quantitative Descriptors

Spectral features: Mel-frequency cepstral coefficients (MFCCs), filter bank energies, and chroma features capture the frequency content and timbre of bowel sounds, which are highly informative for distinguishing sound types and motility patterns [67,73].

Temporal features: Sound intervals, durations, and entropy reflect motility and peristaltic activity. These features are particularly relevant for models assessing bowel motility or detecting abnormal patterns [64,70].

Statistical features: Measures such as mean-crossing rate, spectral bandwidth, and activity scores provide additional information about sound variability and intensity [25,65].

CNNs excel at spectral feature extraction [64,65], while LSTMs and hybrid models handle temporal dynamics [67]. Transformer models incorporate global contextual relevance, outperforming CNNs in limited-data scenarios [68,70].

#### 4.2.3. Machine Learning and Deep Learning Models

Machine learning and deep learning models play complementary roles in the analysis of bowel sounds and the detection of colorectal cancer (CRC), with (a) tabular models and gradient boosting methods excelling in structured data analysis, and (b) convolutional neural network (CNN)-based spectrogram models leading in bowel sound-based signal interpretation and automated bowel sound recognition.

(a) Tabular and Gradient Boosting

These models—including random forests, XGBoost (e.g., XGBoost v1.6–1.7), and stochastic gradient boosting—are highly effective for analyzing structured clinical, laboratory, and epidemiological data relevant to CRC risk and diagnosis. They use hand-engineered features (e.g., lab values, symptom codes, demographic data) and can integrate additional biomarkers such as stool miRNA or CEA levels. Recent studies show that gradient boosting models (e.g., XGBoost) achieve high diagnostic accuracy (AUC up to 0.97) for CRC detection, outperforming traditional biomarkers and enabling risk stratification even in CEA- or FOBT-negative patients [74,75,76]. These models are also useful for identifying key predictive features (e.g., anemia, change in bowel habit) and supporting clinical decision-making in primary care and screening settings [75]. However, their utility in direct bowel sound analysis is limited unless bowel sound-based features are first extracted and tabulated.

(b) CNN-Based Spectrogram Models

CNNs and related deep learning architectures are the state-of-the-art for analyzing raw or minimally processed bowel sound recordings. By converting audio signals into spectrograms or extracting features like Mel-frequency cepstral coefficients, CNNs can automatically learn complex spatial and spectral patterns associated with gastrointestinal motility and pathology. CNNs remain state-of-the-art for bowel sound-based signal analysis. Studies show accuracies up to 93% with high specificity (>97%) for bowel sound classification [64,68,69,70]. These models enable non-invasive, automated assessment of bowel activity, which may support early detection of GI disorders, including CRC, especially as part of multimodal screening strategies [25,64,69,70].

Recent clinical studies and meta-analyses show that convolutional neural network (CNN)-based AI models, especially when integrated with multimodal stool biomarkers, achieve higher diagnostic accuracy for early colorectal cancer (CRC) detection than traditional biomarkers such as carcinoembryonic antigen (CEA) or fecal occult blood testing (FOBT/FIT). However, direct head-to-head comparisons with CNN-based bowel sound analysis remain limited, and most evidence supports multimodal AI approaches rather than acoustic-only models.

(c) Multimodal AI-enhanced stool tests:

A large multicenter study presented at evaluated a noninvasive stool-based test combining mRNA expression, FIT, and an AI/ML algorithm. This approach achieved a sensitivity of 92.3% for CRC and 82.2% for advanced adenomas, with a specificity of 90.1%—substantially outperforming traditional FIT or CEA alone for early-stage disease and precancerous lesions [77]. These results are consistent with other recent advances in multitarget stool DNA/RNA tests, which reach sensitivities above 90% for CRC and 43–46% for advanced polyps, compared to lower sensitivity for FIT and very limited sensitivity for CEA (pooled sensitivity for CEA is only 46%) [78,79].

Similarly, machine learning models such as gradient boosting and random forest, when integrating CEA, FOBT, and other laboratory features, reach AUCs up to 0.97 for CRC detection and can identify CRC even in biomarker-negative patients [76,80,81].

Meta-analyses confirm that AI-enhanced models consistently improve sensitivity and specificity for CRC and advanced polyp detection compared to single-marker tests. Pooled sensitivity and specificity for ML models are 83–92% and 80–91%, respectively, with overall AUROC around 0.88–0.97, whereas FIT and CEA alone have lower sensitivity (FIT: 67–81% for CRC, 23–29% for advanced adenomas; CEA: ~46–60%) [80,82,83]. Combining multiple biomarkers and clinical features in AI frameworks also improves detection in populations with negative single-marker results and enhances risk stratification [76,82,84]. Table 1 summarizes performance metrics.

(d) AI models with traditional biomarkers:

Gradient boosting and other machine learning models using clinical and laboratory data (including CEA, FOBT, and additional features) have demonstrated AUCs up to 0.97 for CRC detection, outperforming CEA and FOBT alone and identifying CRC even in biomarker-negative patients [76,81]. Meta-analyses confirm that machine learning models (including CNNs) consistently improve sensitivity and specificity for CRC and advanced polyp detection compared to traditional single-marker approaches [80,85].

**Table 1:** Sensitivity and specificity of various colorectal cancer (CRC) detection modalities, including CNN-based multimodal stool test, multitarget stool DNA/RNA, FIT/FOBT, CEA serum test, and CNN-based bowel sound analysis.

Modality/Model	Sensitivity for CRC	Sensitivity for Advanced Adenoma	Specificity	Real-World Utility/Notes	Clinical Status
CNN-based multimodal stool test (AI) [77,79,86]	92.3%	82.2%	90.1%	High diagnostic accuracy; noninvasive; evaluated in multicenter studies	Investigational (Late-stage validation)
Multitarget stool DNA/RNA (non-AI) [79]	92–94%	43–46%	87–91%	Widely available; guideline-recommended for average-risk screening	Validated (Guideline-recommended)
FIT/FOBT (traditional) [78,86]	67–74%	23–24%	95%	High specificity; lower sensitivity for advanced adenomas	Validated (Guideline-recommended)
CEA (serum) [76]	~46%	N/A	Variable	Poor sensitivity; not recommended for CRC screening	Not recommended for screening
CNN-based bowel sound analysis [81]	Not established	Not established	Not established	Experimental bowel sound-based signal analysis; CRC-specific validation lacking	Investigational (Research stage)

(e) CNN-based bowel sound analysis:

Promising but not validated; small sample sizes and lack of standardized recording limit clinical adoption [55]. The future potential is recognized, but further research is needed before clinical adoption.

(f) Guideline perspective:

NCCN and ACP recommend stool tests and colonoscopy as first-line screening. Bowel sound-based AI is not yet included [86].

(g) Transformer Models

Transformer models are increasingly important in machine learning and deep learning approaches for bowel sound analysis and colorectal cancer (CRC) detection, offering distinct advantages over tabular, gradient boosting, and convolutional neural network (CNN)-based spectrogram models, particularly in capturing complex dependencies and improving accuracy in data-limited scenarios.

In bowel sound analysis, transformer architectures—especially those incorporating self-attention mechanisms—excel at modeling both global and local dependencies in bowel sound-based signals. For example, the Branchformer model combines self-attention and convolutional gating to robustly extract features from bowel sound recordings, outperforming traditional CNNs and LSTMs, especially when labeled data are limited. Self-supervised pre-training further enhances performance by leveraging large unlabeled datasets, making transformer-based models highly effective for automated, non-invasive bowel sound recognition and early GI disorder detection [68,70].

For CRC detection and grading, transformer models have demonstrated superior performance in histopathological image analysis and clinical data extraction. Studies show that transformer architectures improve detection accuracy by 3–4% over leading CNN-based methods for colon carcinoma grading and classification tasks, and ensemble frameworks integrating transformers with decision tree models (e.g., GastroGPT plus decision trees) achieve high accuracy in early-stage CRC screening, risk assessment, and prognosis [87,88,89]. Transformers are also valuable for extracting relevant features from unstructured patient histories and integrating multimodal data.

Tabular and gradient boosting models (e.g., XGBoost, decision trees) are highly effective for structured clinical and laboratory data, offering strong interpretability and high accuracy for CRC risk prediction and screening, but they rely on hand-engineered features and may not capture complex spatial or temporal patterns [74,76].

CNN-based spectrogram models are state-of-the-art for automated feature extraction from bowel sound-based and image data, excelling in bowel sound event detection and histopathology image classification, but may be limited in modeling long-range dependencies or integrating multimodal information [64,66,69,90].

Transformer models surpass both approaches in handling sequential, multimodal, and unstructured data, providing improved accuracy, robustness, and explain ability, especially when combined with ensemble strategies or self-supervised learning [70,87,88,89]. Importantly, the majority of AI architectures described have been evaluated on non-CRC datasets, small cohorts, or surrogate motility endpoints rather than clinically validated CRC outcomes, limiting direct translational interpretation.

### 4.3. Performance Metrics

Large-scale CRC screening trials guide methodology for future bowel sound AI studies.

#### 4.3.1. Key Methodological Frameworks Include

Multicenter, prospective cohort design: Trials such as the COLOFUTURE and eAArly DETECT studies collect data from multiple clinical sites and diverse populations to ensure generalizability and minimize selection bias. This approach is critical for robust validation and should be adopted for bowel sound studies [77].

Reference standard comparison: AI models are validated against gold-standard diagnostic methods (e.g., colonoscopy and pathology) to determine sensitivity, specificity, and overall accuracy for CRC and advanced adenoma detection. Bowel sound analysis studies should similarly use colonoscopy-confirmed diagnoses as the reference [77,80,91].

Standardized data acquisition and annotation: High-quality, reproducible data collection protocols are essential. For stool and image-based studies, this includes standardized sample handling, imaging protocols, and expert annotation. For bowel sound analysis, uniform recording methods, device calibration, and expert-labeled datasets are needed [55,64,72,92].

#### 4.3.2. Robust Model Development and Validation

Cross-validation and external validation: Models are trained and tested using cross-validation and, crucially, validated on independent external cohorts to assess generalizability and prevent overfitting [80,91,93,94].

Performance metrics: Sensitivity, specificity, AUC, precision, recall, and F1-score are consistently reported. These metrics should be used for bowel sound models to enable fair comparison and clinical relevance [77,80,91,92,93,95].

Handling class imbalance and spectrum bias: Strategies such as stratified sampling, data augmentation, and balanced training sets are employed to address class imbalance and spectrum bias, which can otherwise inflate performance estimates [80,92,94]. Despite encouraging diagnostic performance, direct comparison across AI models remains challenging due to heterogeneity in dataset size, labeling strategies, and validation methodology. CNN-based models often report high accuracy in bowel sound classification; however, many studies rely on relatively small, single-center datasets with potential class imbalance, increasing the risk of overfitting. Transformer-based and hybrid CNN–transformer architectures demonstrate improved robustness in limited-data settings through self-attention and self-supervised pretraining but remain underrepresented in CRC-specific bowel sound-based studies. Importantly, few studies report external validation on independent cohorts, underscoring the need for larger, multicenter datasets and standardized evaluation frameworks to establish generalizability and clinical reliability.

#### 4.3.3. Transparent Reporting and Explainability

Use of explainable AI (XAI): Increasingly, studies incorporate XAI techniques to clarify model decision-making and build clinician trust, especially for clinical integration [93,96,97].

Open data and reproducibility: Calls for publicly available, annotated benchmark datasets and standardized reporting frameworks are common, facilitating reproducibility and fair comparison across studies [72,92].

#### 4.3.4. Clinical Integration and Workflow Assessment

Real-world validation: Some studies assess model performance in routine clinical workflows, not just controlled research settings, to identify practical barriers and optimize implementation [96,97,98].

To guide CNN-based bowel sound analysis studies:

Adopt multicenter, prospective designs with standardized recording and annotation.
Use colonoscopy-confirmed diagnoses as the reference standard.
Employ robust cross-validation and external validation on independent cohorts.
Report comprehensive performance metrics and address class imbalance.
Incorporate explainable AI and strive for open, reproducible data practices.
Evaluate real-world integration and workflow impact.

## 5. Clinical Implications and Translational Utility

CRC is the third most common cancer worldwide. The USPSTF recommends initiating screening at age 45 instead of 50 due to earlier onset trends [99]. Modeling studies show that screening from 45 to 75 years increases life-years gained and reduces mortality [100]. It is critical to distinguish between validated colorectal cancer screening tools and emerging, investigational technologies. Colonoscopy, fecal immunochemical testing (FIT), and multitarget stool DNA/RNA assays are supported by large prospective trials and guideline endorsement, with established performance metrics and defined clinical pathways. In contrast, AI-based bowel sound analysis remains an early-stage research concept, with no CRC-specific prospective validation studies and no established diagnostic accuracy metrics. Current evidence supports its role as an exploratory adjunct for physiological signal analysis rather than a replacement for validated screening modalities.

### 5.1. The Traditional Screening Modalities Approved for CRC Screening

Stool-based screening tests represent the least invasive and most feasible population-level approaches for colorectal cancer (CRC) screening. The fecal immunochemical test (FIT) detects occult blood in stool using antibodies specific to human hemoglobin. Multitarget stool DNA testing (mt-sDNA/FIT-DNA/Cologuard) combines FIT with the detection of DNA biomarkers associated with colorectal cancer and advanced adenomas, offering higher sensitivity for CRC and advanced adenomas than FIT alone but at the cost of lower specificity [101]. High-sensitivity guaiac-based fecal occult blood testing (HSgFOBT) is another stool-based option; however, it is less sensitive and specific than FIT and requires dietary restrictions, unlike FIT. The USPSTF recommends annual HSgFOBT or FIT and mt-sDNA testing every 1–3 years for average-risk screening [102].

Invasive screening tests, particularly colonoscopy, remain the most effective modality for CRC detection due to direct visualization and the ability to perform biopsy and therapeutic intervention in a single procedure [103]. The USPSTF recommends colonoscopy as follow-up for abnormal results from stool-based tests, CT colonography, or flexible sigmoidoscopy [102]. Despite its superior diagnostic performance, colonoscopy is associated with risks such as bleeding and bowel perforation [100]. Flexible sigmoidoscopy offers a less invasive alternative with reduced preparation and faster recovery but does not visualize the proximal colon [104]. Screening recommendations include colonoscopy every 10 years or flexible sigmoidoscopy every 5 years, with the option of flexible sigmoidoscopy every 10 years combined with annual FIT [102].

Radiographic non-invasive tests include colon capsule endoscopy and CT colonography. Colon capsule endoscopy enables visualization of the gastrointestinal tract via an ingestible video capsule, while CT colonography provides three-dimensional imaging of the colon and rectum. Although less invasive than colonoscopy, CT colonography requires bowel preparation and demonstrates reduced sensitivity for flat lesions or polyps smaller than 6 mm [103]. Current USPSTF guidelines recommend CT colonography every 5 years for CRC screening [102].

Blood-based screening tests remain limited. EpiproColon, which detects circulating methylated Septin 9 (SEPT9), is the only FDA-approved blood-based CRC screening test [104]. Reported sensitivity and specificity across all CRC stages are 90% (95% CI, 77.4–96.3%) and 88% (95% CI, 79.6–93.7%), respectively [105]. Emerging blood-based biomarkers, including microRNA and plasma-based assays, are under investigation but are not yet established for routine screening [106,107]. Table 2, Table 3 and Table 4 summarize USPSTF screening recommendations, intervals, and test characteristics.

### 5.2. Bowel Sounds as Physiological Indicators

Bowel sounds reflect peristaltic movement and luminal interaction [108]. MMC phases have documented correlation with bowel sound patterns [30] suggesting diagnostic potential. However, auscultation is limited by variability, noise, lack of standardized criteria, and poor inter-observer agreement [13,108,109,110].

### 5.3. AI Integration into CRC Screening

Artificial intelligence (AI) has increasingly been integrated into healthcare and has demonstrated value across multiple diagnostic domains, including colorectal cancer (CRC), particularly in image interpretation, histopathology, and biomarker-based risk stratification. While traditional screening modalities such as colonoscopy remain highly accurate, their invasive nature, cost, and accessibility barriers limit universal uptake. AI-based approaches have therefore been explored as adjunctive tools to enhance detection efficiency and patient adherence. In CRC management, machine learning algorithms have shown high sensitivity and specificity in recognizing premalignant polyps when applied to validated data sources such as colonoscopy imaging and stool-based biomarkers [77].

In contrast, AI-based bowel sound analysis remains investigational. Although conceptually appealing as a non-invasive and low-cost physiological signal, current evidence does not support its use as a validated screening modality. Existing studies are limited by small sample sizes, non-CRC endpoints, and the absence of prospective clinical validation. Accordingly, bowel sound analytics should be regarded as hypothesis-generating and exploratory rather than as a replacement for established screening tools.

#### Remote and Home-Based Screening Feasibility

Remote and home-based CRC screening is already well established through stool-based modalities, including fecal immunochemical testing (FIT) and multimodal stool biomarker assays. These tests are specifically designed for home use, allowing patients to collect samples without bowel preparation or clinic visits. FIT is recommended annually, while multitarget stool DNA (mt-sDNA) or RNA (mt-sRNA) testing is recommended every three years for average-risk individuals, with reported sensitivities for CRC ranging from 74 to 91% for FIT and 92–94% for mt-sDNA/mt-sRNA, and specificities between 87 and 94% [86].

Recent multicenter studies demonstrate that multimodal AI-enhanced stool tests further improve diagnostic performance, achieving CRC sensitivity of 92.3%, advanced adenoma sensitivity of 82.2%, and specificity of 90.1% [77]. These stool-based approaches are endorsed by the National Comprehensive Cancer Network and the American Gastroenterological Association as first-line options for remote CRC screening [111,112]. In contrast, AI-based bowel sound analysis has not yet achieved sufficient clinical validation to support home-based screening use and remains outside current guideline recommendations.

### 5.4. AI-Based Bowel Sound Analysis

AI-based bowel sound analysis is not yet validated for home screening due to limited clinical data [68,113,114]. No guideline or clinical trial currently supports AI-based bowel sound analysis as a remote screening tool for CRC. Further research is needed before it can be considered alongside FIT or stool biomarker tests [115].

### 5.5. Implementation and Adherence

**Figure 2:**
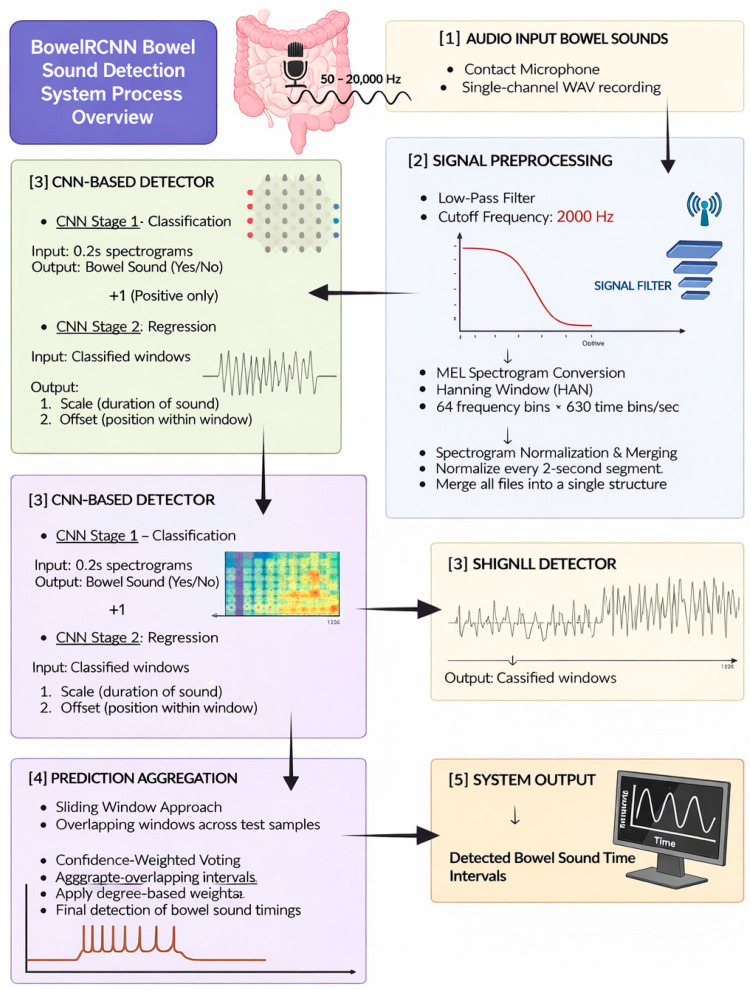
Workflow of the BowelRCNN system for automated bowel sound detection in CRC screening.

**Figure 3:**
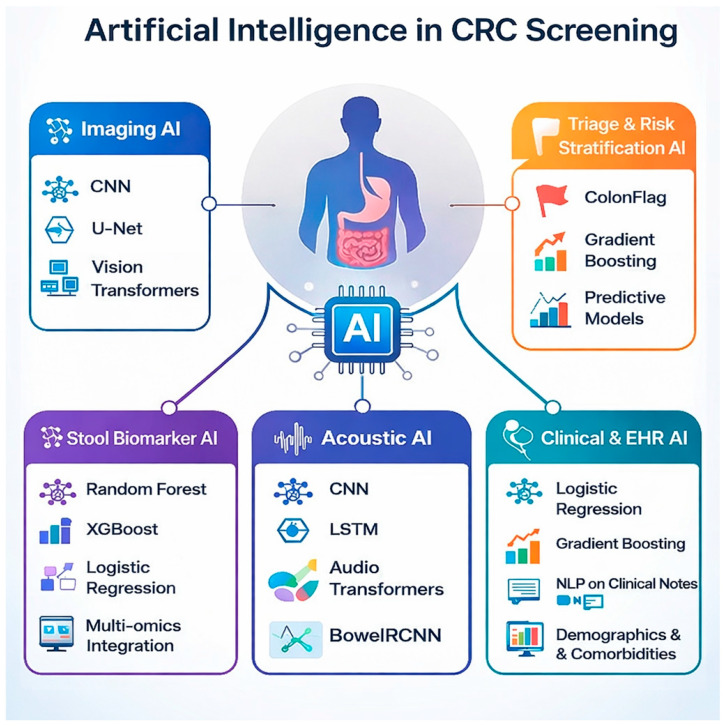
Artificial intelligence supports colorectal cancer (CRC) screening by using imaging, stool biomarkers, bowel sound-based signals, and risk-stratification models to detect disease earlier and more accurately.

**Figure 4:**
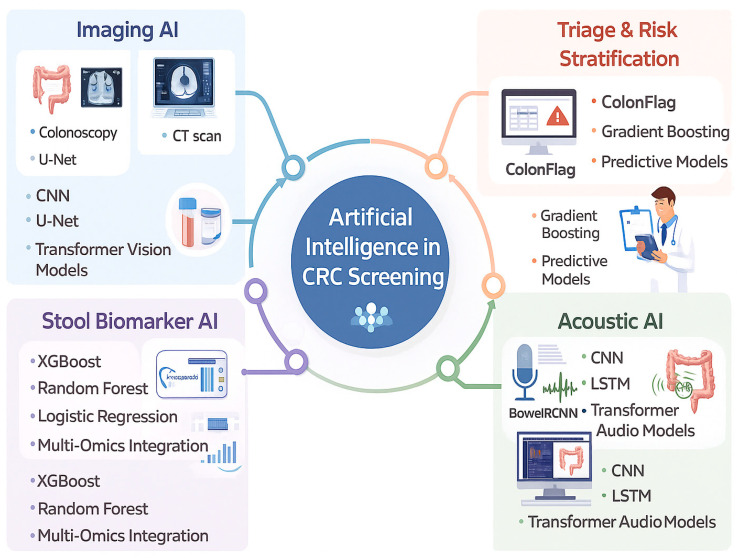
AI enhances colorectal cancer screening by analyzing images, stool biomarkers, bowel sound-based signals, and clinical risk factors to detect disease earlier and more accurately.

Beyond bowel sound analysis, artificial intelligence has demonstrated clinical utility in colorectal cancer screening pathways through integration with established diagnostic modalities. AI-based risk stratification tools, such as ColonFlag, have been shown to enhance diagnostic performance when combined with fecal immunochemical testing in patients undergoing urgent colorectal cancer evaluation. Additionally, meta-analytic evidence suggests that artificial intelligence–assisted colonoscopy significantly improves colorectal neoplasia detection rates among patients with positive fecal immunochemical test results. From an implementation perspective, health economic modeling suggests that artificial intelligence–assisted colonoscopy may represent a cost-effective strategy when deployed as either a primary or secondary screening approach within population-based colorectal cancer screening programs [116,117,118]. Advanced deep learning approaches, including stacking transformer architectures combined with explainable artificial intelligence frameworks, have demonstrated high diagnostic performance for colorectal cancer classification, underscoring the potential of transparent AI models to support clinical decision-making [119]. The convenience of determining gut pathophysiology through bowel sound analysis, combined with substantial advancements in AI, led to a study that developed two smartphone-based AI models. These models utilized the built-in microphone in the smartphone to record and analyze bowel sounds [120]. The study developed two models: the CNN model and the LSTM model. The CNN model demonstrated better accuracy in bowel sound analysis compared to the LSTM model because it was specifically adjusted to work efficiently with the recorded sounds. The CNN model’s moderate accuracy of 83.9% reflects the adequacy of the built-in microphone for sound input that can be analyzed and studied by the AI model [64]. Colorectal cancer tissue produces photoacoustic bowel sound-based signals, which represent the tissue’s ability to convert the laser pulses targeted at it into distinct sound waves. In a study, researchers demonstrated that colorectal cancer tissue produces bowel sound-based signals with weaker amplitudes and altered frequency profiles compared to healthy tissue [121]. Machine learning models processed these signals and achieved high accuracy in distinguishing malignant from normal tissue. Although these bowel sound-based signals differ from natural bowel sounds, the principle of classifying tissue pathology based on bowel sound-based features, including amplitude, frequency spectrum, and energy, demonstrates that the bowel sounds contain detailed information, and combining these kinds of features with AI could help in screening and detecting colorectal cancer. With proper patient data, we may be able to further train AI models to recognize changes in bowel sound patterns, like shifts in frequency or intensity, that are linked to the pre-cancerous stage and cancer [122]. Figure 2, Figure 3 and Figure 4 give an overview of AI in CRC screening and diagnostics.

## 6. Ethical and Regulatory Considerations

### 6.1. Algorithmic Transparency and XAI

Algorithmic transparency and explainable artificial intelligence (XAI) represent central ethical and regulatory considerations in the deployment of AI for colorectal cancer (CRC) screening. Many AI systems—particularly deep learning models—function as “black boxes,” in which the internal decision-making process is not readily interpretable by clinicians or patients. This opacity can undermine trust, complicate clinical accountability, and hinder informed consent, especially when diagnostic errors or unexpected outcomes occur [123,124,125,126].

In CRC screening, the inability to explain AI-driven recommendations is particularly problematic, as clinicians must justify diagnostic decisions and communicate risks and benefits to patients. Lack of interpretability may negatively affect clinician–patient relationships and compromise patient autonomy, reinforcing concerns around transparency and responsibility in clinical care [123,126,127].

Explainable AI (XAI) approaches aim to address these challenges by providing interpretable insights into model behavior. Techniques such as SHAP, LIME, and Grad-CAM are increasingly used to identify feature importance and visualize decision pathways, thereby enhancing transparency and clinical interpretability [124,125,128,129,130]. By enabling clinicians to understand and validate AI outputs, XAI supports trust, facilitates integration into clinical workflows, and aligns with emerging regulatory expectations [124,125,126].

From an ethical and regulatory perspective, transparent and explainable models are essential for clinician acceptance and for meeting legal standards of due diligence and liability [131,132,133,134,135]. Transparency also plays a critical role in informed consent, as patients must understand the role, capabilities, and limitations of AI in their care—an objective that is difficult to achieve with opaque systems [123]. Additionally, lack of interpretability can obscure algorithmic bias, potentially exacerbating health disparities; XAI techniques offer tools to detect and mitigate such biases [132,136]. Reflecting these concerns, emerging regulatory frameworks increasingly emphasize documentation of model interpretability, external validation, and clinician oversight in AI deployment [124,126,132,133].

### 6.2. Data Privacy and Security

Data privacy and security represent critical ethical and regulatory considerations in the application of artificial intelligence (AI) to colorectal cancer (CRC) screening. Protecting patient information, ensuring robust informed consent, and maintaining compliance with regulatory standards are essential for responsible AI deployment.

Patient privacy and confidentiality are paramount, as AI systems require access to large volumes of sensitive health data, including imaging, pathology, and electronic health records. Risks include unauthorized access, data breaches, and inadvertent disclosure of identifiable information. Effective safeguards—such as encryption, secure data storage, and strict access controls—are necessary to prevent exposure of protected health information (PHI) [137,138,139,140].

Informed consent for data use is equally critical. Patients must be clearly informed about how their data are collected, stored, used, and protected, particularly for secondary purposes such as AI model development and training. Transparent consent processes should specify data de-identification practices and potential data sharing or reuse. Inadequate consent and limited patient understanding remain major barriers to ethical AI adoption [123,137,138,141,142].

Compliance with regulatory frameworks is mandatory. In the United States, the Health Insurance Portability and Accountability Act (HIPAA) governs PHI protection, while the European Union’s General Data Protection Regulation (GDPR) and the emerging EU Artificial Intelligence Act impose stringent requirements related to data governance, transparency, and patient rights. AI systems must therefore incorporate data minimization, auditability, and provisions for data access and erasure to meet regulatory expectations [139,143,144,145].

Robust data governance and accountability structures are essential to ensure secure and ethical data use. Clear policies should define data ownership, stewardship, and responsibility for security, supported by centralized repositories, regular audits, and well-defined accountability across clinicians, institutions, and AI developers [141,144,146,147]. Finally, privacy and security frameworks must be coupled with efforts to mitigate algorithmic bias and promote equity, as unrepresentative datasets and opaque data practices can exacerbate health disparities. Inclusive data collection and transparent data handling are therefore fundamental to fair and equitable AI deployment [136,140].

### 6.3. Algorithmic Bias and Medico-Legal Implications

Algorithmic bias represents a significant ethical concern in the application of artificial intelligence (AI) to colorectal cancer (CRC) screening. Bias can arise when training datasets are unrepresentative or reflect existing societal inequities, leading to systematic errors that disproportionately affect racial, ethnic, or socioeconomically marginalized populations. In the context of CRC screening, such bias may result in under-detection or misclassification in certain groups, potentially exacerbating disparities in cancer outcomes. Addressing these risks requires diverse and representative training datasets, subgroup-specific performance evaluation, continuous post-deployment monitoring, and stakeholder engagement throughout the AI lifecycle [136,148,149,150].

Medico-legal implications further complicate AI deployment in CRC screening. Current regulatory frameworks often lag technological advances, creating ambiguity regarding liability when AI-assisted recommendations contribute to adverse clinical outcomes. Responsibility may be unclear among clinicians, healthcare institutions, and AI developers, particularly when opaque “black box” models limit auditability and explanation of decision-making processes. These challenges complicate accountability, informed consent, and post hoc review in cases of diagnostic error or harm [133,134,147,151,152].

Algorithmic transparency and explainable artificial intelligence (XAI) are therefore essential to mitigate both bias and medico-legal risk. Transparent and interpretable models enable clinicians and patients to understand how decisions are generated, support trust, and facilitate regulatory oversight. Explainability techniques such as SHAP and LIME are increasingly recommended to clarify model reasoning and support clinical validation, although their adoption in CRC screening remains variable. Reflecting these concerns, emerging regulatory guidance emphasizes comprehensive model documentation, disclosure of training data characteristics, and clear communication of algorithmic limitations and potential biases [149].

Data privacy and security remain closely intertwined with bias and accountability considerations. AI systems require access to large volumes of sensitive health data, raising risks of breaches, unauthorized use, and secondary misuse. Robust encryption, de-identification practices, and strict data governance frameworks are essential to protect patient information and ensure compliance with regulations such as HIPAA and GDPR. Failure to adequately safeguard data can undermine public trust and expose institutions to legal and ethical liability [132,140,141,146,153].

## 7. Limitations

The future of artificial intelligence (AI) in colorectal cancer (CRC) detection and management is closely linked to the development of large, standardized, and interoperable datasets. Robust multicenter databases are essential for training, validating, and benchmarking AI models, ensuring generalizability and minimizing bias across diverse populations and healthcare settings. Fragmented, single-institution datasets limit model performance and hinder clinical translation, making data harmonization and cross-institutional collaboration a critical priority [114,154,155,156].

Standardization of data acquisition, annotation, and reporting protocols is equally important for reproducibility, regulatory approval, and cross-platform comparison. Uniform standards spanning imaging, pathology, biomarker assays, and clinical metadata facilitate interoperability and enable meaningful evaluation of AI tools across studies. Federated learning frameworks and secure cross-center data-sharing platforms offer promising strategies to address data scarcity and privacy concerns while supporting model robustness and ethical compliance [93,154,155,156,157].

Multicenter validation represents a key step in bridging the gap between proof-of-concept studies and real-world clinical implementation. Prospective, multicenter trials—such as those evaluating AI-enhanced stool biomarker assays—have demonstrated feasibility and diagnostic accuracy in diverse populations. However, continued efforts are required to confirm performance across routine clinical workflows and healthcare systems. Regulatory agencies increasingly mandate external validation, transparent reporting, and clearly defined performance metrics for AI-based medical devices, underscoring the importance of rigorous multi-institutional evidence [77,78,85,158,159].

Integration of AI with molecular biomarkers, multiomics, and liquid biopsy technologies constitutes a major research frontier. AI-driven analysis of circulating tumor DNA, microRNA, and other noninvasive biomarkers supports earlier detection, improved risk stratification, and personalized surveillance strategies. Multimodal data fusion—combining imaging, pathology, genomics, and clinical features—has shown promise in enhancing predictive performance and supporting individualized CRC care [85,154,157,158,160,161,162,163,164].

Wearable devices and telemonitoring systems represent emerging opportunities for remote CRC risk assessment and post-treatment surveillance. AI-enabled wearables can capture physiological, behavioral, and bowel sound-based signals outside traditional clinical environments, potentially improving adherence and enabling longitudinal monitoring. Nevertheless, these technologies require rigorous validation, standardized integration, and alignment with existing care pathways before routine adoption [154,156].

Regulatory pathways for AI in CRC detection continue to evolve. Agencies such as the U.S. Food and Drug Administration increasingly emphasize demonstrations of clinical utility, safety, equity, and external validation, alongside requirements for sensitivity, specificity, and transparent model reporting. Explainable AI (XAI) is gaining prominence to support clinician trust and informed decision-making. Continued collaboration among clinicians, researchers, industry partners, and regulators is essential to address ethical, legal, and practical challenges and to ensure responsible and equitable deployment of AI technologies in CRC care [78,93,97,114,156,157,158,159,165,166].

Finally, the real-world deployment of bowel sound analytics presents additional challenges, including inter-individual anatomical variability, ambient noise, patient posture, and confounding gastrointestinal conditions such as irritable bowel syndrome or postoperative ileus. These factors necessitate robust preprocessing pipelines, standardized recording protocols, anatomically informed acquisition strategies, and context-aware AI models to ensure reliable performance outside controlled research settings.

## 8. Conclusions

Collaboration between humans and artificial intelligence (AI) as a team enhances healthcare decision-making and outcomes in early colon cancer detection by combining the strengths of clinical expertise with the speed, accuracy, and scalability of AI algorithms. This partnership allows clinicians to leverage AI for real-time lesion detection, risk stratification, and biomarker analysis, while retaining oversight and contextual judgment, leading to improved diagnostic accuracy and patient outcomes [1,5,6,7,93,113,114,119,156,166,167].

Human–AI collaboration in colonoscopy enables endoscopists to detect more adenomas and subtle polyps that might otherwise be missed, standardizes quality across operators, and reduces variability in interpretation [97,113,167]. AI can rapidly analyze large datasets, flag suspicious findings, and provide decision support, but clinicians remain essential for integrating these insights with patient history, physical findings, and shared decision-making. As emphasized by Göndöcs and Dörfler, this symbiotic approach fosters trust, accountability, and optimal use of both human and machine intelligence [156,166].

Explainable AI (XAI) directly addresses the transparency issues of “black box” models by making AI decision processes interpretable and auditable for clinicians and patients. Techniques such as SHAP, LIME, and attention heatmaps clarify which features or data points drive AI predictions, allowing clinicians to understand, validate, and communicate the rationale behind AI-generated recommendations [93,97,125,168,169,170,171]. This transparency is critical for clinical trust, regulatory approval, and ethical deployment, as it enables clinicians to challenge or corroborate AI outputs and supports informed consent.

The creation of a large, high-quality database of bowel sounds is a foundational step for advancing AI development in colorectal cancer screening. Such a database would enable robust training and validation of bowel sound-based AI models, improve generalizability, and facilitate benchmarking across institutions and populations [13,99]. Standardized, expert-labeled datasets are essential for developing reliable algorithms, reducing bias, and supporting multicenter research. Ultimately, this resource could unlock noninvasive, accessible screening modalities, complement established tests and expanding early detection capabilities [13,72]. Future research should prioritize standardized data acquisition protocols, multicenter prospective trials with colonoscopy-confirmed endpoints, and regulatory-aligned validation frameworks. Early engagement with regulatory agencies and incorporation of explainable AI methodologies will be critical to translating bowel sound analytics from experimental research into clinically approved screening adjuncts.

Taken together, recent advances in AI-driven bowel sound-based and physiological analytics—ranging from the development of an unsupervised YOLO-based platform for automatic bowel-sound detection and characterization [68,172], to emerging machine-learning frameworks for bio signal interpretation in gastrointestinal diagnostics [173], and the establishment of standardized phonoenterogram metrics to evaluate AI-powered bowel-sound platforms [174,175]—collectively highlight a rapidly evolving landscape in which noninvasive, signal-based tools are poised to transform early colorectal cancer screening and clinical decision-making.

## Figures and Tables

**Table 2:** USPSTF Colorectal Cancer Screening Guidelines (2021 Update)—Age-specific recommendations for CRC screening, corresponding grades of recommendation, net benefits, and clinical notes for each age group.

USPSTF Colorectal Cancer Screening Guidelines (2021 Update)				
Age Group	Recommendation	Grade	Net Benefit	Notes
45–49 years	Recommend screening for colorectal cancer	B	Moderate	Based on increasing incidence of CRC in this age group; modeling shows life-years gained by starting at 45.
50–75 years	Recommend routine screening for colorectal cancer	A	Substantial	Strongest evidence supports screening here; multiple methods are effective.
76–85 years	Clinicians should selectively offer screening	C	Small	Consider overall health, prior to screening history, and patient preferences when making decisions. Benefits are more likely in those who have never been screened.
>85 years	Do not recommend screening	—	Harms outweigh benefits	Competing mortality risks; harms from colonoscopy increase with age.

**Table 3:** Recommended Colorectal Cancer Screening Methods and Intervals—Overview of various CRC screening tests, their types, recommended screening intervals, and important considerations.

Recommended Colorectal Cancer Screening Methods and Intervals			
Screening Methods	Type	Recommendation Interval	Notes
Colonoscopy	Direct visualization	Every 10 years	Highest effectiveness; allows biopsy/removal during same session; requires bowel preparation, sedation.
FIT (Fecal Immunochemical Test)	Stool-based	Every year	Single sample; performed at home; no dietary restrictions.
High-sensitivity guaiac fecal occult blood test (HSgFOBT)	Stool-based	Every year	Requires 3 samples and dietary restrictions; less accurate than FIT; more false positives.
Stool DNA-FIT (sDNA-FIT)	Stool-based	Every 1 to 3 years	Higher sensitivity than FIT but more false positives; entire bowel movement collected.
CT Colonography	Direct visualization	Every 5 years	No sedation: bowel prep required; detects extracolonic findings; follow-up colonoscopy if abnormal.
Flexible Sigmoidoscopy	Direct visualization	Every 5 years	Less invasive than colonoscopy; views only lower colon; fewer life-years gained if used alone.
Flexible Sigmoidoscopy + FIT	Combined strategy	Sigmoidoscopy every 10 years + FIT yearly	Similar benefit to colonoscopy with fewer complications.

**Table 4:** Classification of Colorectal Cancer Screening Tests by Invasiveness and Sensitivity—Categorization of common CRC screening tests according to their invasiveness and relative sensitivity for detecting colorectal neoplasia.

Category	Test Name	Invasiveness	Sensitivity
Stool-Based	FIT	No	High
Stool-Based	mt-sDNA/Cologuard	No	Higher
Invasive	Colonoscopy	Yes	Highest
Invasive	Sigmoidoscopy	Semi	Moderate
Radiographic	CT Colonography	No	Moderate
Radiographic	Capsule Endoscopy	No	TBD
Blood-Based	EpiproColon	No	90%

## Data Availability

This review was based on publicly available academic literature databases.
